# Association of the PhoQ/PhoP Stress Response System with the Internalization of *Escherichia coli* O157:H7 in Romaine Lettuce

**DOI:** 10.3390/microorganisms13020232

**Published:** 2025-01-22

**Authors:** Fnu Chenggeer, Guolu Zheng, Azlin Mustapha

**Affiliations:** 1 Food Science Program, University of Missouri, Columbia, MO 65211, USA; xcmq2@missouri.edu; 2 Cooperative Research Program, Lincoln University, Jefferson City, MO 65102, USA

**Keywords:** *Escherichia coli* O157:H7, internalization, genetic traits, romaine lettuce

## Abstract

Foodborne illness associated with *Escherichia coli* O157:H7 (*E. coli* O157) and romaine lettuce is a serious and persistent food safety issue. This study investigated the ability and associated genetic traits of five *E. coli* O157 strains—namely 86-24, 93-111, C7927, MF1847, and 505B—to internalize in romaine lettuce grown in soil and hydroponic systems. The results showed significant variations in the strains’ ability to internalize, with soil cultivation being more susceptible to *E. coli* O157 internalization relative to hydroponics. Whole-genome comparisons and an analysis of the five *E. coli* O157 strains revealed insights into the potential genetic traits associated with internalization capacity. A single unique gene, ORF-4296, was found to be present in all four internalizing strains (86-24, 93-111, C7927, and MF1847), but absent in the non-internalizing strain 505B. Immediately downstream of OFR-4296 is the PhoQ/PhoP operon, which regulates the important stress responses of *E. coli* O157. Our data showed that this operon was identical in the four internalizing strains but different in strain 505B. Specifically, the C-terminal of PhoQ in strain 505B had a distinct amino acid sequence. The inability of 505B to internalize may be linked to its lack of ORF-4296 and its distinctive C-terminal sequence of PhoQ.

## 1. Introduction

Lettuce food safety is critically important because this vegetable is typically consumed raw and is a frequent carrier of foodborne pathogens, such as pathogenic *Escherichia coli*, *Salmonella* spp., and *Listeria monocytogenes* [1,2]. Romaine lettuce contaminated with *E. coli* O157 was identified as the major cause of 29 foodborne illness outbreaks linked to leafy greens in the United States and Canada during 2009–2018, accounting for at least 13 of the outbreaks [3]. Reducing *E. coli* O157 contamination during the growing, handling, and distribution of lettuce can help prevent illness outbreaks, protect public health, and mitigate associated economic losses.

*E. coli* O157 contamination can occur at any point in the lettuce production and supply chain [4,5]. Research has shown that pre-harvest contamination is critical in foodborne illness outbreaks linked to fresh produce, underscoring the need for preventive measures on farms [6,7,8]. Several pre-harvest environmental factors contribute to introducing foodborne pathogens onto lettuce, including contaminated irrigation water [9,10,11], manure-based soil amendments [12,13], and wildlife intrusions [14,15,16]. At its worst, *E. coli* O157 can internalize in the edible portions of lettuce, significantly increasing the risk of foodborne diseases. Internalized pathogens, shielded by plant tissues, are more resistant to post-harvest washing and sanitation procedures [1,17,18,19,20,21].

The internalization of *E. coli* O157 in lettuce has been reported to be influenced by both abiotic and biotic factors. Abiotic factors, such as sunlight [22,23] and dryness [2,24], were found to reduce the pathogen’s survival on lettuce. Additionally, applying irrigation water above the lettuce has been shown to increase the risk of *E. coli* O157 internalization through leaf apertures, such as stomata [25], and water-saturating soil may contribute to its internalization [26]. However, limited research has explored the risk of *E. coli* O157 internalization in hydroponically grown lettuce, despite the growing popularity of hydroponics.

Biotic factors, including both lettuce and *E. coli* O157 genotypes, contribute to pathogenic internalization. Studies indicated that different lettuce cultivars influenced the internalization of *E. coli* O157 in roots [26,27,28]. Furthermore, recent research has shown that insect-damaged [29] and physically damaged [21,29,30] lettuce is more susceptible to this internalization. The presence of the Shiga toxin genes, *stx*1 and *stx*2, in *E. coli* strains does not affect internalization in lettuce [26], while curli genes may be critical for initiating internalization on leafy green surfaces [31]. However, further investigation is needed to understand the genetic mechanisms underlying *E. coli* O157 internalization in lettuce.

The objectives of this study were to investigate the effects of growth media (soil vs. hydroponic) on *E. coli* O157 internalization in lettuce and to explore, through whole-genome comparisons, the potential genetic traits of *E. coli* O157 that may influence its internalization in lettuce. The findings aim to enhance our understanding of the genetic mechanisms underscoring *E. coli* O157 internalization in lettuce.

## 2. Materials and Methods

### 2.1. Bacterial Strains and Growth

Five strains of *E. coli* O157:H7, *viz*., 86-24, 93-111, 505B, C7927, and MF1847, were obtained from the Food Microbiology Laboratory at the University of Missouri, Columbia, Missouri. Tryptic Soy broth (TSB) (Becton Dickinson and Company, Sparks, MA, USA) was used for the routine culture (overnight without agitation) of the bacteria at 37 °C. The monitoring of the amount of *E. coli* O157 was carried out by spread-plating on MacConkey sorbitol (SMAC) agar (Becton Dickinson and Company, Sparks, MD, USA).

For the spiking of *E. coli* O157 into the soil or hydroponic growth systems, 9 mL of freshly grown overnight culture was centrifuged at 10,062× *g* at 4 °C for 10 min and was washed twice in 9 mL of PBS (MP Biomedicals, Solon, OH, USA) to remove any remaining nutrients from the TSB. The final bacterial concentration of this *E. coli*–PBS suspension was about 10^7^ CFU/mL.

### 2.2. Growing Lettuce

A class II type A/B3 biosafety cabinet (NuAire Inc., Plymouth, MN, USA) was used as a lettuce growth chamber for this study. The lettuce was grown in soil or hydroponic media without recirculation and aeration at room temperature, as detailed in Section 2.3. Three sets of 10-Watt LED lights (Walmart Inc., Bentonville, AR, USA) were used to provide light for 12 h per day, and the height of the lights was adjusted accordingly with the growth of the lettuce. The humidity was maintained at a minimum of 45% by covering the lettuce with plastic bags. Upon germination, the plants were fertilized once a week by adding a mixture of Grow Big 6-4-4 (Foxfarm, Arcata, CA, USA) and calcium–magnesium supplement 1-0-0 (General Hydroponics, Sebastopol, CA, USA), according to the manufacturer’s recommendation, to maintain the nutrient level. The mixture was filtered through a 0.22 µm membrane (Millipore, Burlington, MA, USA) and stored at room temperature before use.

### 2.3. Comparison of the Internalization of E. coli O157 in Lettuce Grown in Soil vs. in a Hydroponic System

#### 2.3.1. Growing Lettuce in Soil vs. in a Hydroponic System, with the Spiking of *E. coli* O157

To grow lettuce in soil, ten plastic pots (9 cm in diameter × 10 cm in height) were filled with approximately 150 g of potting mix (Miracle-Gro Co., Marysville, OH, USA) and placed individually in a 500 mL plastic beaker to avoid any potential cross-contamination among pots via irrigation water. The entire set of pots with beakers was autoclaved at 121 °C for 15 min before seeding. In each pot, five to ten seeds (Paris White Cos, Morgan County Seeds LLC, Barnett, MO, USA) were planted at a depth of about 0.5 in below the soil surface. Sterile deionized water (400 mL) was periodically added into the beakers to moisten the soil, and 1.5 mL of the aforementioned *E. coli*–PBS suspension (around 10^7^ CFU/mL) was added to the soil around the seeds in each pot (duplicate pots per strain). The bacterial concentration in the treatment group was monitored every seven days, starting from when the lettuce germinated (day 0), by spread-plating 100 µL of the solution in the beakers onto SMAC agar plates. The bacterial population was maintained at >10^3^ CFU/mL during the experiment by adding the *E. coli*–PBS suspension periodically to the soil whenever the *E. coli* concentration decreased to or below 10^3^ CFU/mL.

Once the lettuce germinated, the two sturdiest seedlings were kept in each pot, and the others were removed. Fertilizing was carried out, as aforementioned in Section 2.2.

In parallel, 10 beakers (250 mL) were used for the hydroponic growth of lettuce. Rock wool cubes (5 × 5 × 5 cm) (Halatool Co, Xuzhou, Jiangsu, China) were autoclaved and used as a seed holder. Five to ten lettuce seeds were placed on a rock wool cube and transferred inside the aforementioned beaker filled with 150 mL of a hydroponic growth broth, Grow Big^®^, (Foxfarm Co., Arcata, CA, USA), allowing the bottom of the wool rock to soak in the nutrient broth. Quotas of 1.5 mL of the aforementioned *E. coli*–PBS suspension were added to each beaker. A piece of foil paper was wrapped around the top of the beaker to prevent the evaporation of the broth. Once the lettuce germinated, the two best-growing plants were kept in the same manner as for the soil system. The pH of the growth broth was kept between 6.5 and 7.5, adjusting as needed. The plant was fertilized once a week, as was the case for the soil system.

The above experiments were conducted three times.

#### 2.3.2. Detection of *E. coli* Internalization in Lettuce

Five lettuce leaves from each pot were harvested by aseptic cutting on days 20 (D20), 30 (D30), and 40 (D40) after the generation of seeds (day 0), respectively. The leaves were dipped in a 10% bleach solution for 5 s [30], while the cut openings were kept away from the sanitizer. The surface-sanitated portions of leaves were then rinsed by dipping and swirling in four containers containing 300 mL of sterile deionized water. After air drying for 5 min in the biosafety cabinet to remove visible water, the leaves were pressed in a sterile bag to extract the juice. If juice was lacking, appropriate amounts of PBS were added to facilitate homogenization. The lettuce juice (100 µL) was spread-plated onto SMAC agar and the plates were incubated at 37 °C for up to 24 h to look for the presence or absence of *E. coli* O157 in the samples.

The colonies that morphologically appeared to be *E. coli* O157 on the SMAC agar plates were serologically confirmed through a serological agglutination test using an *E. coli* O157 Latex kit (Oxoid, Basingstoke, Hants, UK), following the manufacturer’s instructions.

In total, 45 leaf samples each from soil-grwon and hydroponically grown lettuce were examined for the presence or absence of each *E. coli* O157 strain. The internalization frequency of each strain was calculated for D20, D30, and D40 samples by dividing the number of observed internalizations in the three bacterial spiking experiments conducted (Section 2.3.1) by three (3).

### 2.4. Whole-Genome Sequencing and Comparisons of E. coli O157

To investigate the genetic traits of *E. coli* O157 that are potentially involved in their ability to internalize in lettuce, bioinformatic approaches were used, based on whole-genome comparisons.

#### 2.4.1. Genome DNA Extraction and Whole-Genome Sequencing

Overnight cultures of the five *E. coli* O157 strains were used to extract their genomic DNA using the UltraClean Microbial Kit (Qiagen, Hilden, Germany), following the manufacturer’s instructions. The concentration and purity of the isolated DNA were measured using a NanoDrop spectrophotometer (Thermo Fisher Scientific, Wilmington, DE, USA). The DNA samples were then sent to Azenta Life Sciences (Burlington, MA, USA) for whole-genome sequencing, using Illumina’s next-generation sequencing (NGS) and the paired-end approach with 30× coverage.

#### 2.4.2. Whole-Genome Comparisons

The genetic distance of the five *E. coli* O157 strains was determined through the online program NDtree 1.2 [32] using the resulting raw reads of each strain from whole-genome sequencing. The raw reads of each strain were also assembled to generate contigs for each strain using the online assembly program Shovill [33], which is available at the GalaxyTrakr website (https://galaxytrakr.org, accessed on 21 December 2024). GalaxyTrakr is a part of Galaxy, an open platform assisting users in the analysis of genomic data for foodborne pathogens. The gene makeups of each strain were then determined using the online program GeneMarkS-2 [34], with the aforementioned contigs. The resulting gene makeups of each strain were subjected to pairwise comparisons using the online BLAST program available at the National Center for Biotechnology Information (https://www.ncbi.nlm.nih.gov).

## 3. Results

### 3.1. Variations in the E. coli O157 Internalization in Lettuce

In this study, an *E. coli* O157 strain was considered capable of internalizing in lettuce when it was detectable in the juice of the surface-sanitized leaves. As shown in Table 1, all strains but 505B were detectable from the lettuce cultivated in the soil, and fewer strains were detectable from the hydroponic system, where only strains 86-24, C7927, and MF1847 were detectable. Therefore, strain 505B was never detectable, and strain 93-111 was detectable in the soil system but not in the hydroponic one. Additionally, more strains were more often detectable at D40 from the soil system than from the hydroponic system, although the detectable frequency was one-third. Strain MF1847 was detectable in the samples of D20, D30, and D40 from both systems.

### 3.2. The Open Reading Frames (ORFs) Associated with E. coli O157 Internalization

**Figure 1:**
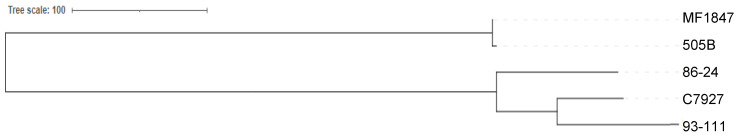
Phylogenetic tree of the *E. coli* O157:H7 strains based on their genetic distance.

**Figure 2:**
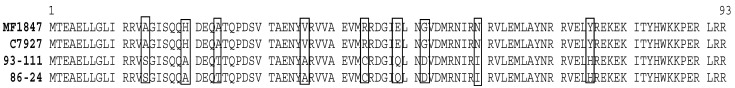
Amino acid (AA) sequences of ORF-4296 in the four internalization strains. The AA variations are highlighted by a rectangle.

**Figure 3:**
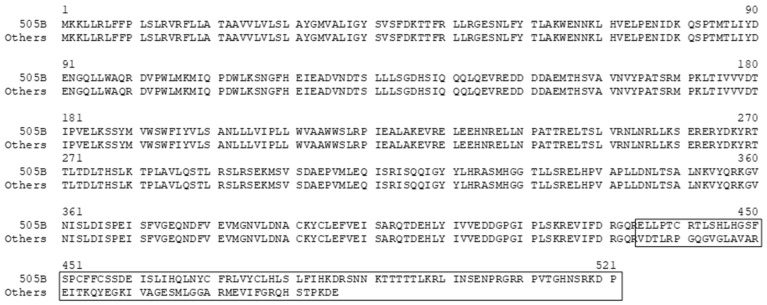
Variations in the C-terminal of PhoQ. The differences in AA sequences and lengths are highlighted by a rectangular box. Others refer to the four internalization strains.

The genetic-distance analysis indicated that among the five *E. coli* O157 strains, strains 93-111 and 505B were genetically the most distant, while strains MF1847 and 505B were the closest (Table 2, Figure 1). The gene makeup, determined by GeneMarkS-2, demonstrated that the five *E. coli* O157 strains had a significant variation in the number of ORFs and that strains MF1847 and 505B contained the closest numbers of ORFs (Table 3), which was in alignment with their genetic distance. Further analysis via the pairwise whole-genome comparisons showed that MF1847 had all the ORFs that were found in 505B, and that MF1847 contained a single unique ORF—ORF-4296 (arbitrarily assigned by GeneMarkS-2)—which was absent in 505B; however, some ORFs of the two strains appeared different in copy numbers and amino acid sequences. In the other three internalized strains, C7927 contained an ORF identical to MF1847′s ORF-4296, while a similar ORF, differing by nine amino acids, was found in strains 93-111 and 86-24 (Figure 2). ORF-4296 is 93 amino acids in length (Figure 2) and is known to be a hypothetical protein conserved in *E. coli,* according to the GenBank^®^. Immediately upstream of ORF-4296 is a phage-packing-related protein and the direct downstream ORF codes for a PhoQ protein, a two-component system sensor histidine kinase, belonging to the PhoP-PhoQ operon containing seven ORFs (genes). Furthermore, the seven genes of the PhoP-PhoQ operon were identical in all four internalizing strains. In contrast, the non-internalizing strain, 505B, showed differences in amino acid (AA) sequence and molecular size, being 35 AAs longer, in the C-terminal of PhoQ (Figure 3).

In summary, strains 86-24, 93-111, C7927, and MF1847 were detectable in lettuce, and shared the unique ORF-4296, as well as an identical PhoP-PhoQ operon. In contrast, strain 505B was undetectable in lettuce, lacked ORF-4296, and exhibited differences in the C-terminal region of PhoQ.

## 4. Discussion

Although *E. coli* O157 internalization in lettuce has been documented [26,28], it has not always been observed [35,36]. In our study, the soil cultivation system (an abiotic factor) generally showed a higher frequency of internalization, particularly evident in D40 samples and for strain 93-111, compared to the hydroponic system (Table 1), suggesting that the soil system was generally more susceptible to *E. coli* O157 internalization. A similar observation was made by Franz et al. (2007), where *E. coli* O157 internalization was not detected in lettuce grown hydroponically, but was observed in soil-cultivated vegetables, although a higher bacterial dose was spiked into the soil [35]. However, these findings contradict the general conclusion drawn by Hirneisen et al. (2012) regarding the internalization of human enteric pathogens (*E. coli* O157, *L. monocytogenes*, and *Salmonella* spp.) in food crops, including lettuce. After reviewing 23 studies on the subject, Hirneisen et al. (2012) concluded that pathogen-contaminated soil resulted in little to no internalization compared to contaminated hydroponic solutions [37]. This trend held true for *Salmonella* in most cases but not for *E. coli* O157:H7 internalization in lettuce. Notably, Franz’s study was the only one among the 23 that directly compared soil-grown and hydroponically grown lettuce [35]. It is important to note that this study utilized sterilized soil and hydroponic systems, thereby eliminating potential competitive interferences from other microorganisms. Additionally, the cultivation systems did not appear to significantly influence the internalization of strains MF1847 and 505B. Specifically, MF1847 internalization was detectable at all sampling times in both systems, whereas 505B internalization was never detected in either system (Table 1). This suggests that the internalization of *E. coli* O157 was also influenced by bacterial genotypes, highlighting the role of biotic factors in this process.

Mutational analysis is a common method for investigating the functions of bacterial genes [38,39]. Genetically, *E. coli* O157 is a group of *E. coli* strains that evolved via mutations from a common ancestor. MF1847 and 505B (Table 2, Figure 1), the two genetically closest strains with differences in only a limited number of ORFs and the ability to internalize, have provided a novel window to examine potential genes (ORFs) associated with internalization. ORF-4296 was the only gene found through whole-genome comparisons across all four internalizing strains, but was absent in the non-internalizing strain 505B. This suggests that ORF-4296 may be critical for internalization. However, the function of ORF-4296 remains unknown, offering no direct information about its potential role in the internalization. Interestingly, the C-terminal of PhoQ of 505B was found to be different from that of the other four strains (Figure 2), suggesting that 505B may have a different sensing response to environmental factors. The PhoQ/PhoP system is known to play a critical role in regulating the stress adaptation response of *E. coli*, including response to low environmental Ca^2+^ or Mg^2+^, acid tolerance, osmolarity, and toxicity [40,41]. Vegetables, including lettuce, are not the primary habitat of *E. coli* O157; therefore, the vegetable internal environment is stressful for internalized bacteria. It is reasonable to assume that stress adaptation mechanisms are needed for *E. coli* O157 to persist in lettuce and that the PhoQ/PhoP operon is related to the stress adaptation mechanisms. However, it is unknown at this point what stress response is critical in *E. coli* O157 internalization and if ORF-4296 is involved in PhoQ/PhoP regulation in response to environmental stress during internalization.

## 5. Conclusions

This study highlights significant variations in the internalization capabilities of five *E. coli* O157 strains in romaine lettuce, influenced by both cultivation systems (soil vs. hydroponic) and bacterial genetic traits. Strain 505B was consistently undetectable in the edible portion of lettuce from both cultivation systems, while the other four strains exhibited varying internalization frequencies, with soil-based cultivation showing higher susceptibility. The absence of the hypothetical ORF-4296 and the distinct C-terminal sequence of PhoQ in strain 505B suggest that these genetic elements may be involved in *E. coli* O157 internalization. Additionally, the PhoQ/PhoP system, known for regulating stress adaptation, may contribute to the persistence of *E. coli* O157 in lettuce. These findings may provide insights into the genetic mechanisms underlying pathogenic internalization. A better understanding of the mechanisms may lead to developing novel strategies for preventing *E. coli* O157 internalization in lettuce. Therefore, further investigation is needed into the functions of ORF-4296 and the PhoQ/PhoP system in *E. coli* O157 internalization.

## Figures and Tables

**Table 1:** The internalization frequency of *E. coli* O157:H7 strains in a soil (S) system vs. a hydroponic (H) system.

Sampling Time	Internalization Frequency (Frequency of Being Detected in the Triplicate Experiments) by Strain									
	86-24		93-111		505B		C7927		MF1847	
	S	H	S	H	S	H	S	H	S	H
D20	2/3	1/3	2/3	0/3	0/3	0/3	1/3	3/3	1/3	1/3
D30	1/3	2/3	1/3	0/3	0/3	0/3	0/3	2/3	2/3	2/3
D40	1/3	0/3	1/3	0/3	0/3	0/3	1/3	0/3	1/3	1/3

**Table 2:** Genetic distance among the *E. coli* O157:H7 strains.

**Strain**	86-24	93-111	505B	C7927	MF1847
86-24	0	90	370	115	360
93-111	90	0	394	139	393
505B	370	394	0	319	3
C7927	115	139	319	0	318
MF1847	369	393	3	318	0

**Table 3:** Gene makeup of the *E. coli* O157:H7 strains.

Strain	Number of ORFs	Difference in ORF Number
505B	5434	0
MF1847	5466	32
93-111	5643	209
86-24	5743	309
C7927	5847	403

## Data Availability

The original contributions presented in this study are included in the article. Further inquiries can be directed to the corresponding authors.
